# Prognostic Role of Adverse Events in a Worldwide Population of Patients with Biliary Tract Cancer Treated with Chemo-Immunotherapy

**DOI:** 10.3390/cancers18182921

**Published:** 2026-09-09

**Authors:** Linda Bartalini, Margherita Rimini, Masafumi Ikeda, Oluseyi Abidoye, Jessica Lucchetti, Lorenzo Antonuzzo, Jin Won Kim, Arndt Vogel, Angela Lamarca, Federico Nichetti, Anna Saborowski, Tiziana Pressiani, Caterina Vivaldi, Ilario Giovanni Rapposelli, Jeroen Dekervel, Chiara Braconi, David James Pinato, Emiliano Tamburini, Chiara Carlotta Pircher, Stefano Tamberi, Florian Castet, Monica Verrico, Alessandro Parisi, Nuno Couto, Hong Jae Chon, Andrea Martirena, Matteo Landriscina, Fabian Finkelmeier, Ester Oneda, Antonio Avallone, Emanuela Dell’Aquila, Lukas Perkhofer, Il Hwan Kim, Vincenzo Formica, Cidalia Maria de Sousa Pinto, Ingrid Garajova, Beodeul Kang, Salvatore Corallo, Elisabetta Fenocchio, Giovanni Farinea, Alessandro Pastorino, Anna Diana, Changhoon Yoo, Marta Schirripa, Maria Grazia Rodriquenz, Mario Scartozzi, Lucrezia Zumstein, Michele Ghidini, Gerald W. Prager, Giuseppe Aprile, Gian Paolo Spinelli, Yung-Yeh Su, Stephen L. Chan, Emily Warmington, Alessia Lancianese, Tomoyuki Satake, Tanios Bekaii-Saab, Daniele Lavacchi, Laura Passeri, Michele Ferrara, Minsu Kang, Alessandra Anna Prete, Silvia Bozzarelli, Mara Persano, Andrea Pretta, Gianluca Masi, Rita Balsano, Monica Niger, Sara Lonardi, Francesca Salani, Silvia Camera, Lorenzo Fornaro, Andrea Casadei-Gardini, Lorenza Rimassa

**Affiliations:** 1 Unit of Medical Oncology 2, Azienda Ospedaliero-Universitaria Pisana, 56124 Pisa, Italy; lindabarta92@gmail.com (L.B.); caterina.vivaldi@unipi.it (C.V.); gianluca.masi@unipi.it (G.M.); francesca.salani@unipi.it (F.S.); l.fornaro@ao-pisa.toscana.it (L.F.); 2 IRCCS San Raffaele Hospital, 20132 Milan, Italy; 3 Vita-Salute San Raffaele University, 20132 Milan, Italy; 4 Department of Hepatobiliary and Pancreatic Oncology, National Cancer Center Hospital East, Kashiwa 277-8577, Japan; 5 Department of Internal Medicine, Mayo Clinic, Phoenix, AZ 85054, USA; 6 Fondazione Policlinico Universitario Campus Bio-Medico, 00128 Roma, Italy; 7 Clinical Oncology Unit, Careggi University Hospital, 50134 Florence, Italy; lorenzo.antonuzzo@unifi.it (L.A.);; 8 Department of Experimental and Clinical Medicine, University of Florence, 50139 Florence, Italy; 9 Division of Hematology/Medical Oncology, Department of Internal Medicine, Seoul National University Bundang Hospital, Seoul National University College of Medicine, Gumi-ro 173 Beon-gil, Bundang-gu, Seongnam 13620, Republic of Koreaminsu198@snu.ac.kr (M.K.); 10 Department of Gastroenterology, Hepatology, Infectious Disease and Endocrinology, Hannover Medical School, 30625 Hannover, Germany; 11 Division of Gastroenterology and Hepatology, Toronto General Hospital, Medical Oncology, Princess Margaret Cancer Centre, Schwartz Reisman Liver Research Centre, Toronto ON M5G 2C4, Canada; 12 Department of Medical Oncology, FJD Comprehensive Cancer Centre, ENETS Centre of Excellence (GEP and Lung)—Fundacion Jimenez Diaz University Hospital, 28040 Madrid, Spain; 13 Fundación Jiménez Díaz University Hospital, Health Research Institute Fundación Jiménez Díaz (IIS-FJD), Universidad Autónoma de Madrid (UAM), 28040 Madrid, Spain; 14 Oncology 1 Unit, Department of Medical Oncology, Veneto Institute of Oncology IOV—IRCCS, 35128 Padua, Italysara.lonardi@iov.veneto.it (S.L.); 15 Hepatopancreatobiliary Oncology Unit, Humanitas Cancer Center, IRCCS Humanitas Research Hospital, Rozzano, 20089 Milan, Italysilvia.bozzarelli@cancercenter.humanitas.it (S.B.); rita.balsano@cancercenter.humanitas.it (R.B.); 16 Department of Translational Research and New Technologies in Medicine and Surgery, University of Pisa, 56124 Pisa, Italy; 17 Department of Medical Oncology, IRCCS Istituto Romagnolo per lo Studio dei Tumori (IRST) “Dino Amadori”, 47014 Meldola, Italy; 18 Digestive Oncology, University Hospitals Leuven, 3000 Leuven, Belgium; jeroen.dekervel@uzleuven.be; 19 School of Cancer Sciences, University of Glasgow, Glasgow G61 1QH, UK; chiara.braconi@glasgow.ac.uk; 20 Beatson West of Scotland Centre, Glasgow G12 0YN, UK; 21 CRUK Scotland Centre, Glasgow G61 1BD, UK; 22 Department of Surgery&Cancer, Imperial College London, Hammersmith Hospital, London W12 0NN, UK; david.pinato@imperial.ac.uk; 23 Department of Oncology and Palliative Care, Cardinale G. Panico, Tricase City Hospital, 73039 Tricase, Italy; e.tamburini@piafondazionepanico.it (E.T.); pretealessandra@gmail.com (A.A.P.); 24 Department of Medical Oncology, Fondazione IRCCS Istituto Nazionale dei Tumori, 20133 Milan, Italy; 25 Santa Maria Delle Croci Hospital of Ravenna, AUSL Romagna, 48121 Ravenna, Italy; stefano.tamberi@unibo.it; 26 Department of Medical and Surgical Sciences, University of Bologna, 40126 Bologna, Italy; 27 Upper Gastrointestinal and Endocrine Tumor Unit, Vall d’Hebron Institute of Oncology (VHIO), Vall d’Hebron University Hospital, 08035 Barcelona, Spain; 28 UOC Oncologia A, Department of Hematology, Oncology and Dermatology, Policlinico Umberto I, Sapienza University of Rome, 04019 Rome, Italy; 29 Clinica Oncologica e Centro Regionale di Genetica Oncologica, Università Politecnica Delle Marche, Azienda Ospedaliero-Universitaria Delle Marche, 60126 Ancona, Italyalessia.lancianese@ospedaliriuniti.marche.it (A.L.); 30 Digestive Unit, Champalimaud Clinical Centre, Champalimaud Research Centre Champalimaud Foundation, 1400-038 Lisbon, Portugal; 31 Division of Medical Oncology, Department of Internal Medicine, CHA Bundang Medical Center, CHA University School of Medicine, Seongnam 13496, Republic of Korea; minidoctor@cha.ac.kr (H.J.C.);; 32 Department of Medical Oncology, OECI Comprehensive Cancer Centre—ENETS Centre of Excellence (GEP and Lung)—Fundacion Jimenez Diaz University Hospital, Health Research Institute Fundación Jiménez Díaz (IIS-FJD), 28040 Madrid, Spain; andrea.martirena@quironsalud.es; 33 Medical Oncology and Biomedical Therapy, Policlinico Riuniti, 71122 Foggia, Italy; matteo.landriscina@unifg.it; 34 Medical Clinic 1, University Hospital, Goethe-University Frankfurt, 60590 Frankfurt am Main, Germany; 35 Dipartimento di Oncologia medica, Fondazione Poliambulanza, 25124 Brescia, Italy; 36 Clinical Experimental Abdominal Oncology Unit, Istituto Nazionale Tumori-IRCCS Fondazione G. Pascale, 80131 Naples, Italy; a.avallone@istitutotumori.na.it; 37 IRCCS Istituto Nazionale Tumori Regina Elena, 00138 Roma, Italy; 38 Internal Medicine 1, University Hospital Ulm, 89081 Ulm, Germany; 39 Institute of Molecular Oncology and Stem Cell Biology, Ulm University Hospital, 89081 Ulm, Germany; 40 Division of Oncology, Department of Internal Medicine, Haeundae Paik Hospital, Inje University College of Medicine, Busan 48108, Republic of Korea; 41 Oncologia Medica, Dipartimento di Medicina dei Sistemi, Università di Tor Vergata, 00133 Roma, Italy; 42 Department of Medical Oncology, Unidade Local de Saude do Algarve (ULSAlg), 8000-286 Faro, Portugal; cmpinto@ulsalg.min-saude.pt; 43 Medical Oncology Unit, University Hospital of Parma, Parma 43126, Italy; 44 Oncology Unit, Fondazione IRCCS Policlinico San Matteo, 27100 Pavia, Italy; salvatore_corallo@hotmail.it; 45 Department of Internal Medicine and Medical Therapy, University of Pavia, 27100 Pavia, Italy; 46 INOC—Istituto Nazionale Oncologico Candiolo, IRCCS, 10060 Candiolo, Italy; 47 Department of Oncology, University of Turin, San Luigi Hospital, Orbassano, 10043 Turin, Italy; 48 Medical Oncology, IRCCS Ospedale Policlinico San Martino, 16132 Genova, Italy; 49 Oncology Unit, Ospedale del Mare, 80147 Napoli, Italy; 50 ASAN Medical Center, University of Ulsan College of Medicine, Seoul 05505, Republic of Korea; 51 Medical Oncology Unit, Department of Oncology and Hematology, Belcolle Hospital, 01100 Viterbo, Italy; marta.schirripa@asl.vt.it; 52 Oncology Unit, Fondazione IRCCS “Casa Sollievo della Sofferenza, 71013 San Giovanni Rotondo, Italy; mg.rodriquenz@operapadrepio.it; 53 Medical Oncology, University Hospital and University of Cagliari, 09042 Cagliari, Italy; 54 GI Oncology Unit and Familial Cancer Unit, Oncology Department, Hospital Universitario 12 de Octubre, 28041 Madrid, Spain; lucrezia.zumstein@unito.it (L.Z.); andrea.pretta@unica.it (A.P.); 55 Fondazione IRCCS Ca’Granda Ospedale Maggiore Policlinico, 20122 Milan, Italy; michele.ghidini@policlinico.mi.it; 56 Department of Medicine I, Clinical Division of Oncology, Medical University Vienna, 1090 Vienna, Austria; 57 Department of Medical Oncology, AULSS 8 Berica, 36110 Vicenza, Italy; giuseppe.aprile@asufc.sanita.fvg.it; 58 UOC Oncologia Territoriale, Polo Pontino, La Sapienza Università Di Roma, 04100 Latina, Italy; gianpaolo.spinelli@uniroma1.it; 59 National Institute of Cancer Research, National Health Research Institutes, Tainan 704, Taiwan; yysu@nhri.edu.tw; 60 Department of Oncology, National Cheng Kung University Hospital, College of Medicine, National Cheng Kung University, Tainan 701401, Taiwan; 61 Department of Clinical Oncology, Prince of Wales Hospital, the Chinese University of Hong Kong, Hong Kong SAR, China; 62 Oxford University Hospitals NHS Foundation Trust, Oxford OX3 9DU, UK; 63 Department of Biomedical Sciences, Humanitas University, Pieve Emanuele, 20072 Milan, Italy

**Keywords:** biliary tract cancer, cholangiocarcinoma, immune-mediated adverse events, corticosteroids

## Abstract

Background: Biliary tract cancers (BTCs) are aggressive malignancies with limited treatment options. The addition of durvalumab or pembrolizumab to cisplatin–gemcitabine improves overall survival (OS), but validated biomarkers of benefit remain limited. Immune-mediated adverse events (imAEs) have been proposed as treatment-emergent clinical correlates of immune activation. Methods: We retrospectively analyzed an international cohort of patients with locally advanced or metastatic BTC treated with first-line cisplatin–gemcitabine plus durvalumab. Primary endpoints were OS and progression-free survival (PFS). The principal exposure was the occurrence of any imAE. A post hoc complete-case Cox model was adjusted for age, sex, primary tumor site, prior surgery, ECOG performance status, disease stage, albumin, bilirubin, and neutrophil-to-lymphocyte ratio (NLR); analyses of individual AEs were exploratory. Exact AE onset dates were not consistently available, precluding a reliable formal time-dependent exposure analysis. Results: A total of 1358 patients were included; all received durvalumab, and 276 (20.3%) developed at least one imAE. In the fully baseline-adjusted model, any imAE remained associated with a lower risk of death (adjusted HR 0.72, 95% CI 0.57–0.92; *p* = 0.008) and progression or death (adjusted HR 0.63, 95% CI 0.52–0.77; *p* < 0.001). In exploratory baseline-adjusted analyses of individual imAEs, other imAEs remained associated with OS (HR 0.39, 95% CI 0.24–0.62; *p* < 0.001) and PFS (HR 0.56, 95% CI 0.40–0.77; *p* < 0.001), while hypothyroidism remained associated with PFS only (HR 0.65, 95% CI 0.43–0.97; *p* = 0.034); rash and hyperthyroidism were not independently associated after full baseline adjustment. In AE-focused exploratory models, decreased appetite and hyponatremia were associated with worse outcomes, neutropenia with better outcomes, and ALT elevation with worse PFS. Baseline corticosteroid use was uncommon (*n* = 21), and all steroid analyses are vulnerable to confounding by indication and exposure-timing bias. Conclusions: Treatment-emergent imAEs may represent potential prognostic markers or clinical correlates of benefit, but they are not validated surrogate endpoints. Prospective studies with precise AE-onset capture are required.

## Simple Summary

Biliary tract cancers are aggressive malignancies with limited treatment options. Combining chemotherapy with immunotherapy has become the standard first-line treatment for advanced disease, but clinically useful markers of benefit remain limited. We evaluated treatment-emergent adverse events in a large international real-world cohort of 1358 patients receiving cisplatin–gemcitabine plus durvalumab. Immune-mediated adverse events (imAEs) occurred in 20.3% of patients and were associated with longer overall and progression-free survival even after adjustment for major baseline prognostic factors. Because exact imAE onset dates were not consistently available, this observational association may be affected by immortal time bias and should not be interpreted as validation of a surrogate endpoint. Baseline corticosteroid exposure was uncommon (21 patients), making its association with poorer outcomes particularly vulnerable to confounding by indication; corticosteroids administered for imAE management were not significantly associated with worse survival. These findings support imAEs as potential clinical correlates of treatment benefit that require prospective validation with precise exposure timing.

## 1. Introduction

Biliary tract cancers (BTCs) comprise a heterogeneous group of highly aggressive malignancies, including intrahepatic cholangiocarcinoma (iCCA), extrahepatic cholangiocarcinoma (eCCA), and gallbladder carcinoma (GBC) [1,2]. Cholangiocarcinoma was first described by Durand-Fardel in 1840 [3]. Although BTCs are considered rare, their epidemiology is markedly heterogeneous: in an international registry analysis, overall BTC incidence ranged from 1.25 to 14.35 per 100,000 person-years, with the highest incidence reported in Chile, whereas mortality was highest in the Republic of Korea [4]. A single worldwide rank for all BTCs is not provided by GLOBOCAN because the component diseases are coded separately; in 2022, GBC alone accounted for 122,491 new cases and 89,055 deaths worldwide, ranking 22nd for incidence and 20th for cancer mortality [5]. BTC are often diagnosed at an advanced stage, and long-term survival remains poor [1,2]. For advanced disease, the phase III ABC-02 trial established cisplatin–gemcitabine (CG) as a reference regimen, with median OS of 11.7 versus 8.1 months with gemcitabine alone (HR 0.64, 95% CI 0.52–0.80; *p* < 0.001) [6]. The treatment landscape subsequently expanded to include biomarker-driven targeted therapies [7,8] and immune checkpoint inhibitors. In TOPAZ-1, adding durvalumab to CG improved median OS from 11.5 to 12.8 months (HR 0.80, 95% CI 0.66–0.97), while KEYNOTE-966 showed median OS of 12.7 versus 10.9 months with pembrolizumab plus CG versus CG alone (HR 0.83, 95% CI 0.72–0.95) [9,10,11,12,13,14]. A recent meta-analysis of the two randomized trials confirmed an OS benefit for chemo-immunotherapy (pooled HR 0.80, 95% CI 0.72–0.89) [15]. Alongside treatment advances, there is increasing interest in biomarkers capable of refining prognosis and treatment stratification in BTC. Conventional clinical variables such as ECOG performance status, disease extent, albumin, bilirubin, and systemic inflammatory indices remain relevant, and contemporary real-world data also identify poor ECOG performance status and hypoalbuminemia as adverse prognostic factors [16]. Molecular profiling can identify actionable alterations such as *IDH1* mutations, *FGFR2* fusions, and *HER2* amplification, while circulating tumor DNA and other liquid-biopsy approaches are being investigated for non-invasive genomic profiling, response assessment, resistance detection, and recurrence monitoring [16,17]. Emerging tissue-based signatures may add complementary information; for example, *STMN1* expression has recently been associated with prognosis and differential treatment response in BTC [18]. Treatment-emergent clinical events may provide an additional dynamic layer of information. Across solid tumors, immune-mediated adverse events (imAEs) have been associated with immune-checkpoint inhibitor efficacy [19,20,21,22,23], and a dedicated exploratory analysis of TOPAZ-1 reported a numerically greater durvalumab treatment effect among patients with recorded imAEs [24]. At the same time, systemic corticosteroids used to manage immune toxicity raise concerns because of potential immunosuppressive effects. We therefore evaluated the association of imAEs and non-imAEs with OS and PFS in a large international real-world cohort of patients with advanced BTC treated with first-line CG plus durvalumab [25,26,27], while also examining corticosteroid exposure as an exploratory clinical factor.

## 2. Materials and Methods

### 2.1. Study Population

The study population included patients with unresectable, locally advanced, or metastatic BTC, encompassing iCCA, eCCA (distal and perihilar), and GBC. All patients received first-line CG in combination with durvalumab (1358/1358; 100%); no patient received pembrolizumab. Data were retrospectively collected from 55 centers across 12 countries (Italy, Germany, Austria, Spain, Portugal, Belgium, United Kingdom, United States of America, Republic of Korea, China, Hong Kong Special Administrative Region of China, and Japan) from February 2022 through April 2025, with data cutoff on 1 May 2025. Treatment consisted of intravenous CG plus durvalumab every 21 days for up to eight cycles: durvalumab 1500 mg on day 1, gemcitabine 1000 mg/m^2^ on days 1 and 8, and cisplatin 25 mg/m^2^ on days 1 and 8. After completion of CG, durvalumab 1500 mg every 4 weeks could be continued until disease progression or unacceptable toxicity. Adverse events were collected during treatment, and patients were classified according to whether at least one treatment-emergent AE had been recorded.

The following toxicities were evaluated:

- imAEs: pruritus, rash, hypothyroidism, hyperthyroidism, colitis, and other imAEs (musculoskeletal, pulmonary, neurological, cardiac);
- non-imAEs: fatigue, anemia, neutropenia, thrombocytopenia, leukopenia, thrombocytosis, nausea, decreased appetite, ALT elevation, fever, constipation, diarrhea, vomiting, neuropathy, cholangitis, hyponatremia, hypokalemia, insomnia, other non-imAEs.

The present study was approved by the local Ethics Committee at each center, complied with the provisions of the Good Clinical Practice guidelines and the Declaration of Helsinki and local laws, and fulfilled the Regulation (EU) 2016/679 of the European Parliament and of the Council of 27 April 2016 on the protection of natural persons with regard to the processing of personal data.

### 2.2. Statistical Analysis

This work evaluated OS and PFS as primary endpoints and their associations with treatment-emergent imAEs and non-imAEs. OS was defined as the time from initiation of first-line therapy to death from any cause. PFS was defined as the time from initiation of first-line therapy to disease progression or death, whichever occurred first. OS and PFS were estimated using the Kaplan–Meier method, and survival curves were compared using the log-rank test.

Unadjusted hazard ratios (HRs) for any imAE, individual imAEs, and non-imAEs were estimated using Cox proportional hazards models. In the harmonized dataset, AE occurrence was recorded as a binary ever/never variable and exact onset dates were not consistently available. Accordingly, a reliable formal time-dependent exposure model for imAEs or non-imAEs could not be implemented, and these analyses should be interpreted as associative and potentially affected by immortal time bias. The overall any-imAE analysis was the principal exposure analysis. Individual imAE and non-imAE analyses, including two AE-focused multivariable Cox models (one entering overall imAEs and one entering individual imAEs together with non-imAEs that were significant in univariable analysis), were considered exploratory. Corticosteroid use at baseline and corticosteroids administered for imAE management were evaluated in separate exploratory Cox models; the latter is a post-baseline exposure and was therefore interpreted cautiously.

To address confounding by baseline prognosis, a post hoc complete-case Cox sensitivity model was fitted including any imAE together with age, sex, primary tumor location (iCCA vs. eCCA/GBC), prior surgery, ECOG performance status (≥1 vs. 0), disease stage (metastatic vs. locally advanced), albumin (<3.5 vs. ≥3.5 g/dL), bilirubin (elevated vs. normal), and NLR (≥3 vs. <3), irrespective of univariable statistical significance. No missing-data imputation was performed. A sensitivity analysis used robust standard errors, clustered by treatment center. An analogous baseline-adjusted exploratory model assessed individual imAEs. Because a large number of individual AEs were evaluated, no multiplicity correction was applied and *p*-values for individual-event analyses are nominal; borderline associations should be considered hypothesis-generating. Patients were monitored every 2–3 months using multiphasic imaging according to each center’s clinical practice. Response was classified according to RECIST version 1.1. Median follow-up was estimated using the reverse Kaplan–Meier method. AEs were graded according to CTCAE version 5.0. HRs are reported with 95% confidence intervals (CIs) and two-sided *p*-values; *p* < 0.05 was used as a descriptive threshold for statistical significance.

Descriptive and AE-focused survival analyses were performed using MedCalc software (MedCalc, version 16.8.4). Fully baseline-adjusted and center-clustered Cox sensitivity analyses were performed in Python 3.13.5 using statsmodels 0.14.6.

## 3. Results

### 3.1. Characteristics of the Study Population and Development of AEs

Between February 2022 and April 2025, 1358 patients were included in the study; all 1358 (100%) received first-line cisplatin–gemcitabine plus durvalumab. Overall, 711 patients (52.4%) were male, and the median age was 69 years (range 24–91 years). A total of 758 patients (55.8%) had iCCA, 366 (27.0%) had eCCA, and 234 (17.2%) had GBC. Baseline characteristics are summarized in Table 1.

At data cutoff (1 May 2025), the median duration of follow-up was 14.5 months (95% CI: 13.7–36.5); 934 patients (68.7%) discontinued treatment due to disease progression, and 605 patients (44.5%) had died.

**Figure 1:**
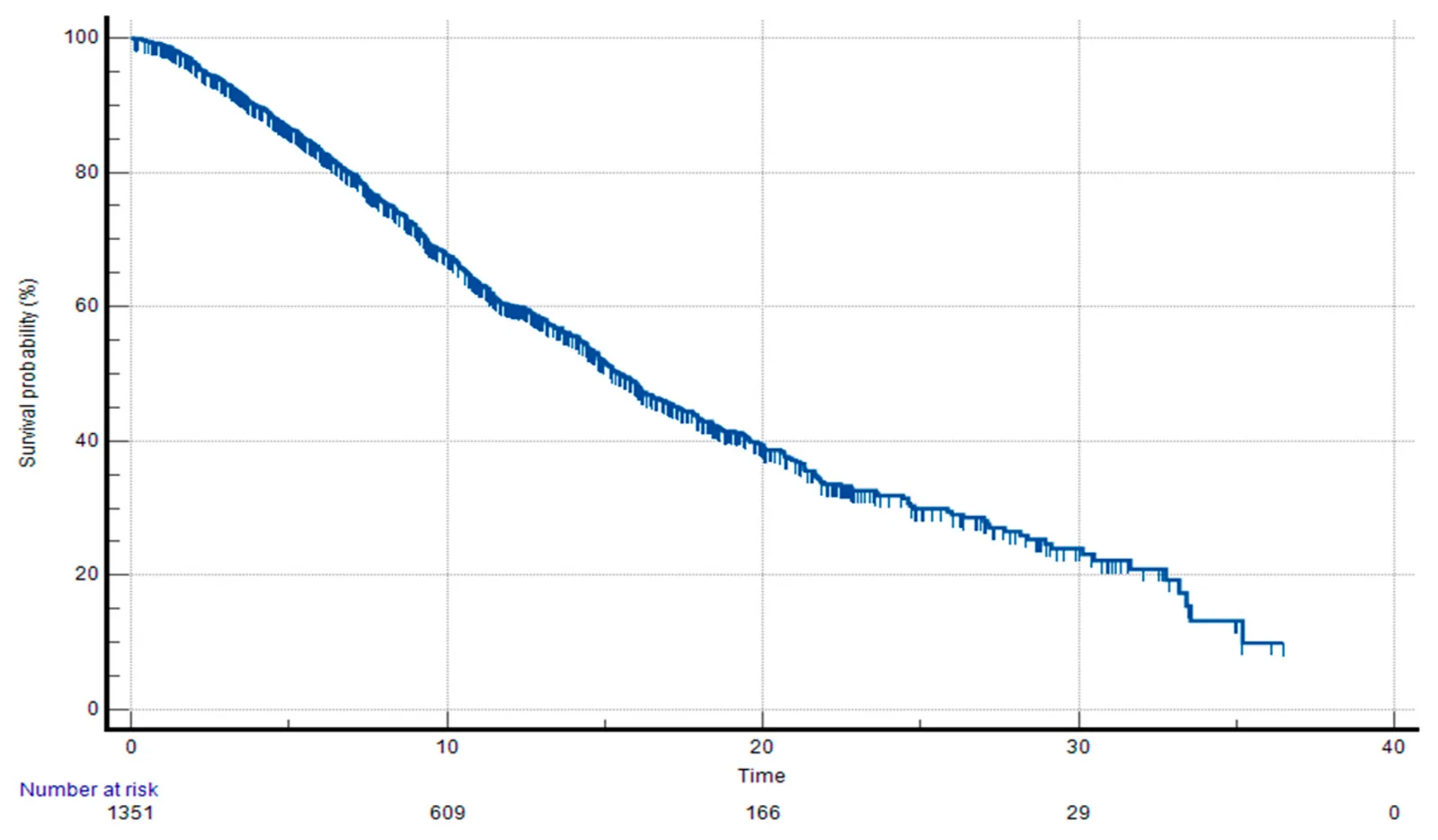
Kaplan–Meier curve for overall survival.

**Figure 2:**
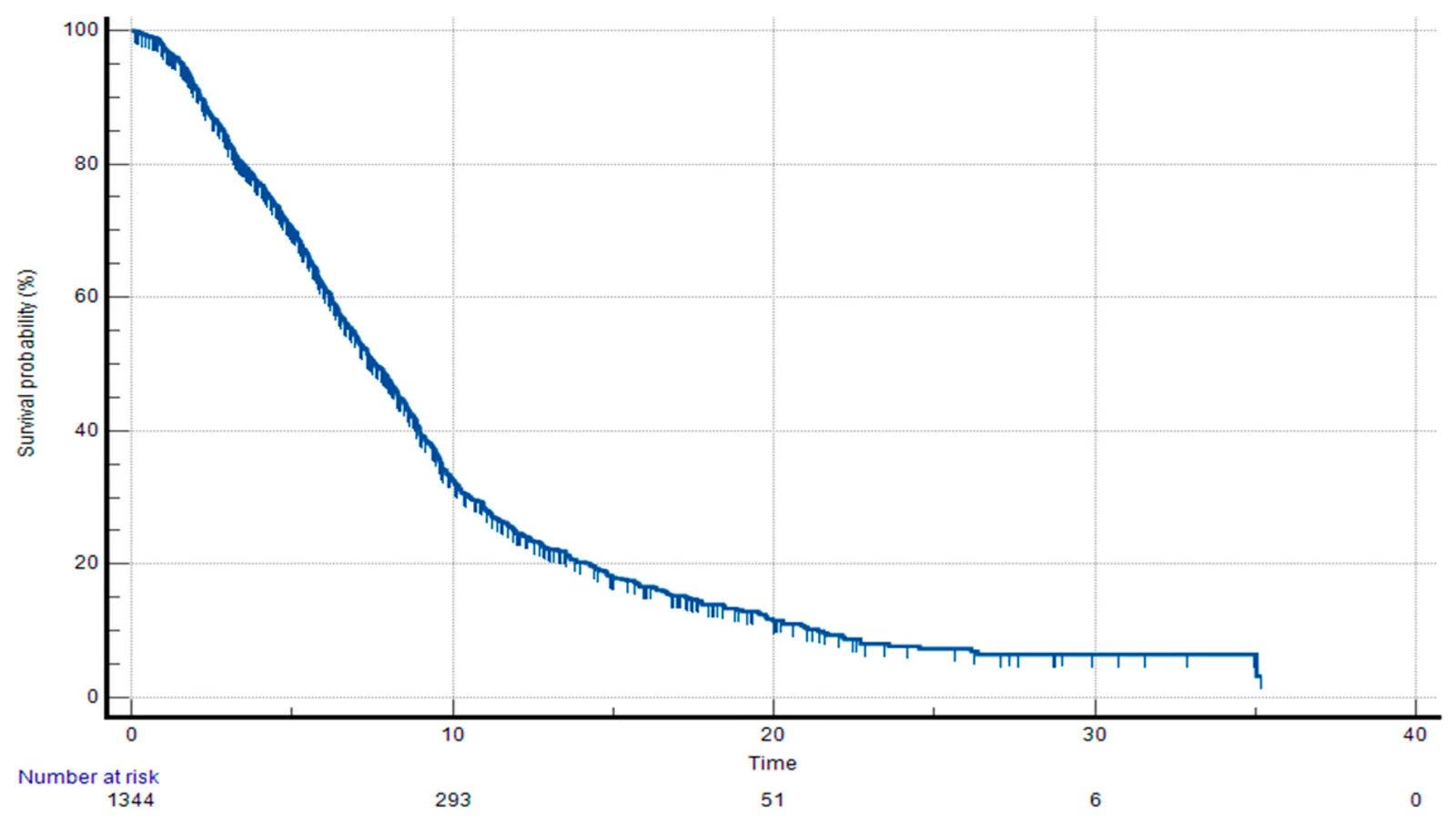
Kaplan–Meier curve for progression-free survival.

Estimated median OS was 15.6 months (95% CI: 14.6–16.5) and median PFS was 7.6 months (95% CI: 7.2–8.1) (Figure 1 and Figure 2).

A total of 276 patients (20.3%) developed imAEs of any grade. Among these 276 patients, the most frequently reported imAEs were pruritus (112, 40.6%) and rash (110, 39.9%); endocrine events included hypothyroidism (65, 23.6%) and hyperthyroidism (27, 9.8%), immune-mediated colitis occurred in 21 patients (7.6%), and other imAEs (musculoskeletal, pulmonary, neurological, or cardiac) were reported in 97 patients (35.1%). Among the full cohort, the most common any-grade non-imAEs were fatigue (706, 52.0%), anemia (634, 46.7%), neutropenia (588, 43.3%), thrombocytopenia (488, 35.9%), leukopenia (419, 30.8%), and thrombocytosis (141, 10.4%). Gastrointestinal toxicities included nausea (408, 30.0%) and decreased appetite (328, 24.1%). Other frequently reported events were ALT elevation (297, 21.9%), fever (257, 18.9%), neuropathy (157, 11.6%), hyponatremia (117, 8.6%), and hypokalemia (98, 7.2%) (Table 2). Patients could experience more than one AE. Most AEs were grade 1–2, whereas grade 3–4 events were less frequent. Among imAEs, 235 patients (17.3% of the full cohort) experienced grade 1–2 events and 40 (2.9%) experienced grade 3–4 events. Individual imAEs were predominantly low-grade, with grade 3–4 rash, pruritus, and colitis occurring in only three (0.2%), one (0.1%), and one (0.1%) patient, respectively. Grade 3–4 non-imAEs were more frequent among hematological toxicities, particularly neutropenia (*n* = 286, 21.1%), thrombocytopenia (*n* = 102, 7.5%), anemia (*n* = 78, 5.7%), and leukopenia (*n* = 78, 5.7%) (Table 3).

### 3.2. Survival Analysis

**Figure 3:**
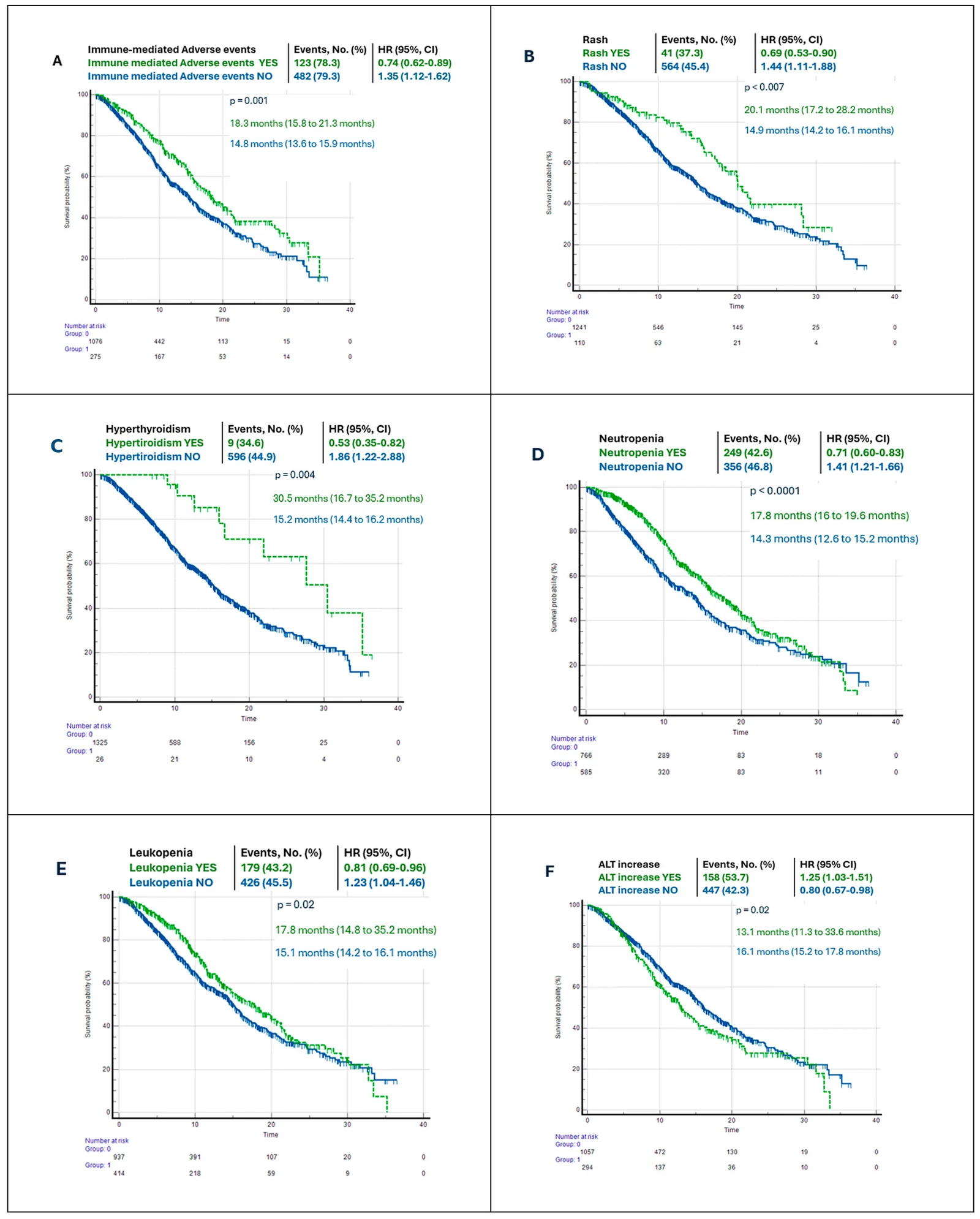

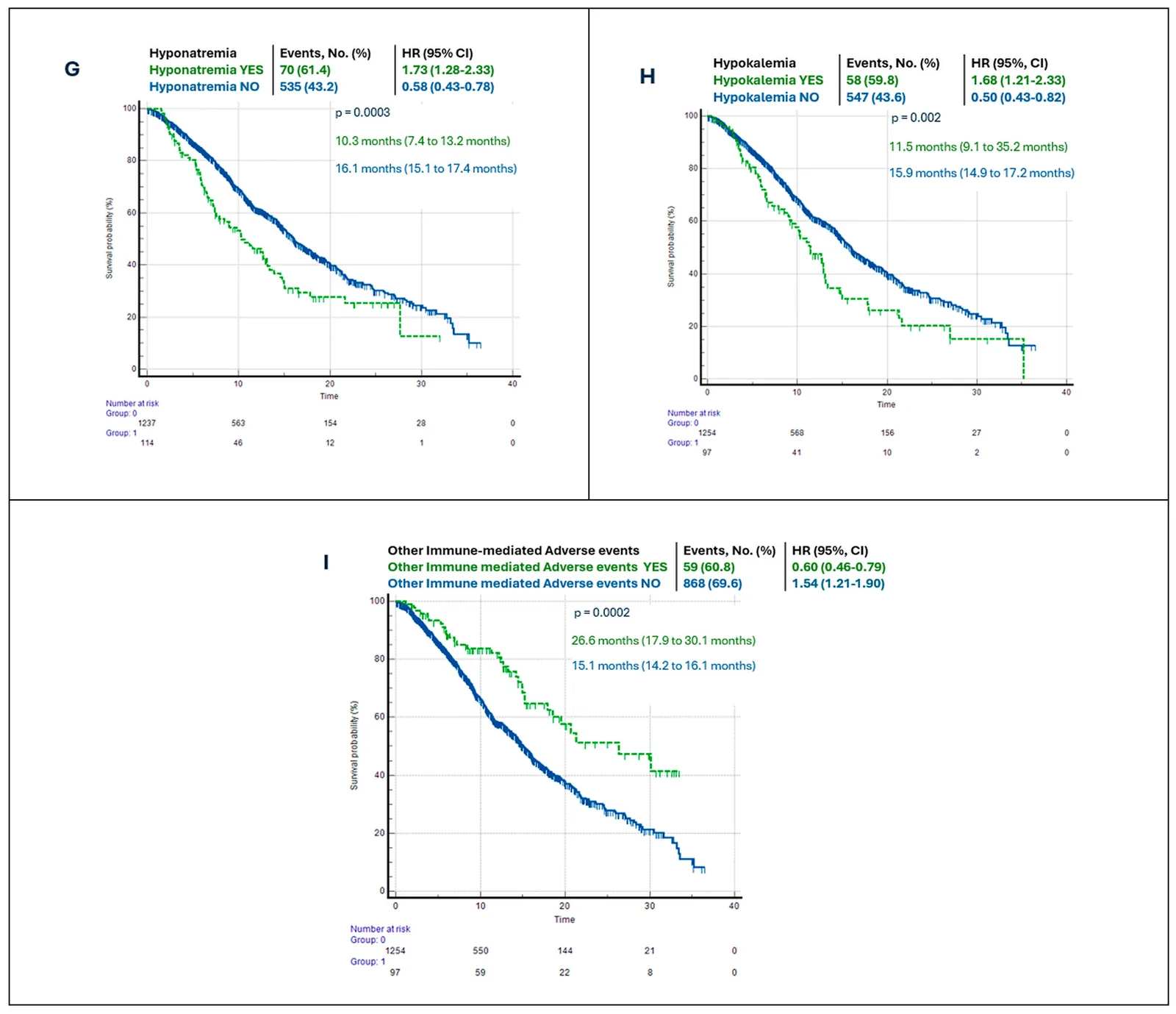
(**A**) Kaplan–Meier curve of OS comparing patients who developed immune-mediated adverse events (imAEs) versus those who did not; (**B**) Kaplan–Meier curve of OS comparing patients who developed rash to those who did not; (**C**) Kaplan–Meier curve of OS comparing patients who developed hyperthyroidism events to those who did not; (**D**) Kaplan–Meier curve of OS comparing patients who developed neutropenia to those who did not; (**E**) Kaplan–Meier curve of OS comparing patients who developed leukopenia to those who did not; (**F**) Kaplan–Meier curve of OS comparing patients who developed ALT increase to those who did not; (**G**) Kaplan–Meier curve of OS comparing patients who developed hyponatremia to those who did not; (**H**) Kaplan–Meier curve of OS comparing patients who developed hypokalemia to those who did not; and (**I**) Kaplan–Meier curve of OS comparing patients who developed other imAEs to those who did not.

In exploratory univariable analyses of OS according to individual AEs (Table 4), imAEs overall (HR 0.74, 95% CI 0.62–0.89; *p* = 0.001), rash (HR 0.69, 95% CI 0.53–0.90; *p* = 0.007), hyperthyroidism (HR 0.53, 95% CI 0.35–0.82; *p* = 0.004), and other imAEs (HR 0.60, 95% CI 0.46–0.79; *p* = 0.0002) were associated with lower risk of death. Among non-imAEs, neutropenia (HR 0.71, 95% CI 0.60–0.83; *p* < 0.0001) and leukopenia (HR 0.81, 95% CI 0.69–0.96; *p* = 0.02) were associated with better OS, whereas ALT elevation (HR 1.25, 95% CI 1.03–1.51; *p* = 0.02), decreased appetite (HR 1.58, 95% CI 1.32–1.94; *p* < 0.0001), hyponatremia (HR 1.73, 95% CI 1.28–2.33; *p* = 0.0003), and hypokalemia (HR 1.68, 95% CI 1.22–2.33; *p* = 0.002) were associated with worse OS. No significant association with OS was observed for the other AEs evaluated (Figure 3).

**Figure 4:**
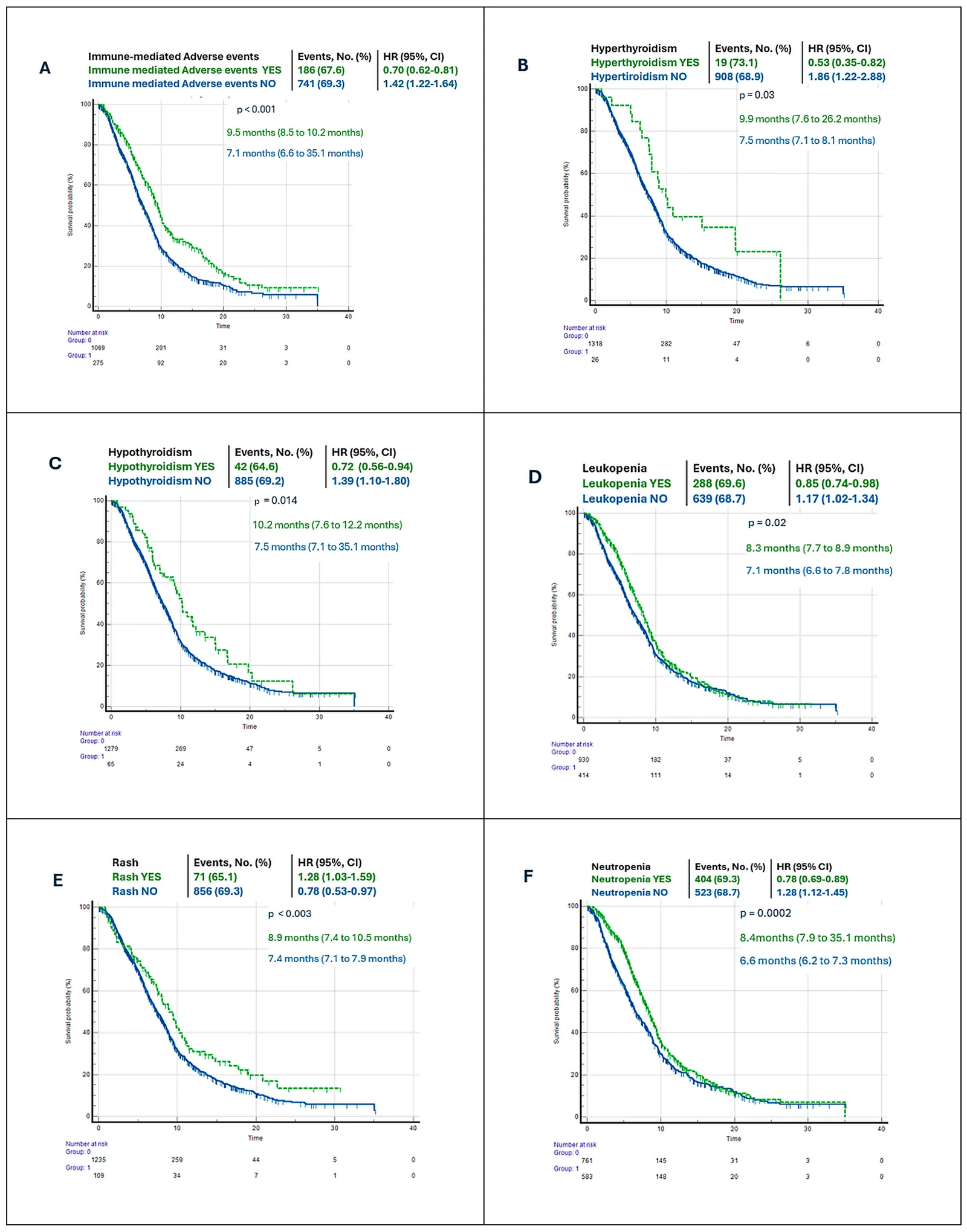

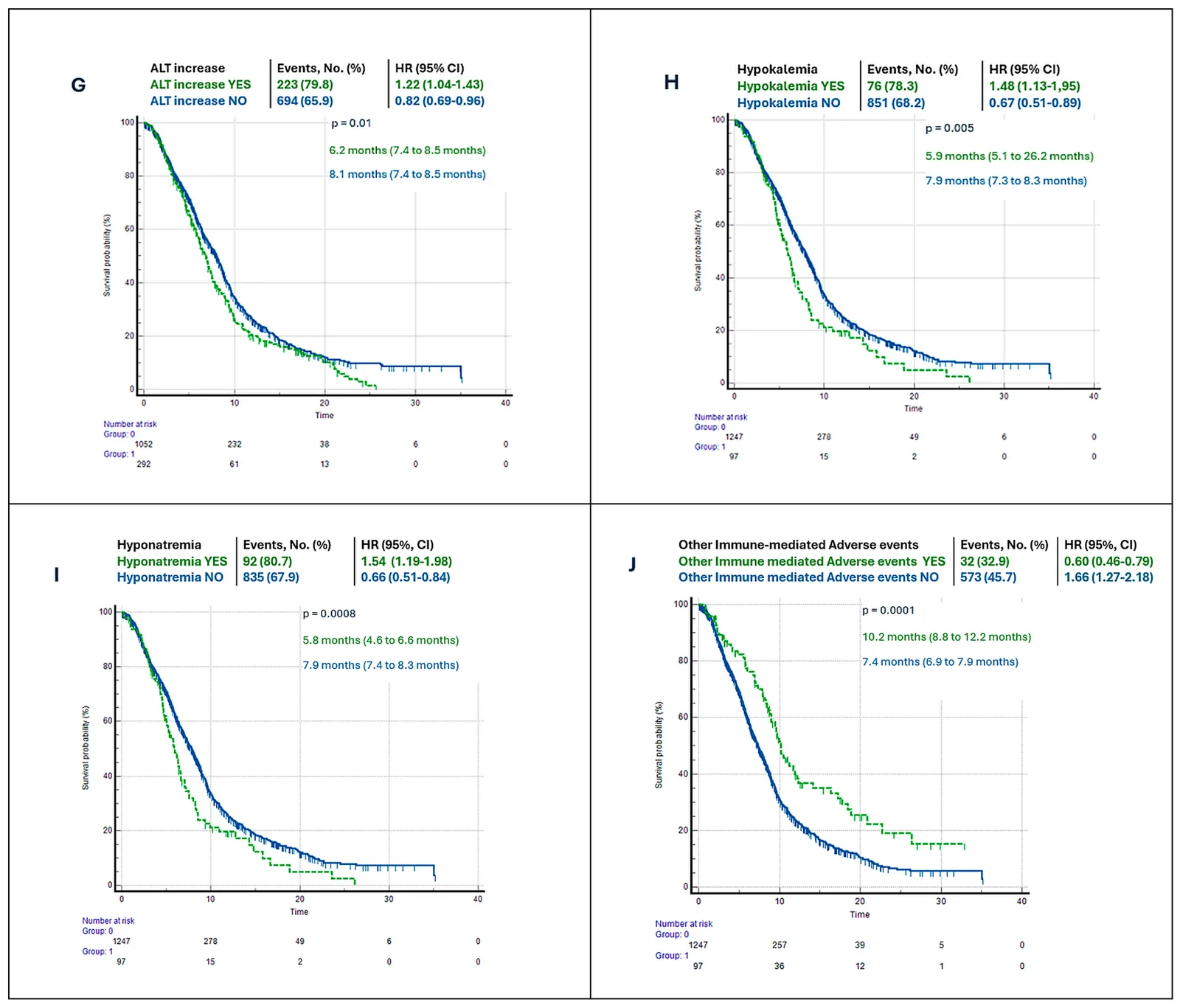
(**A**) Kaplan–Meier curves of progression-free survival (PFS) comparing patients who developed immune-mediated adverse events (imAEs) to those who did not; (**B**) Kaplan–Meier curve of PFS comparing patients who developed hyperthyroidism to those who did not; (**C**) Kaplan–Meier curve of PFS comparing patients who developed hypothyroidism to those who did not; (**D**) Kaplan–Meier curve of PFS comparing patients who developed leukopenia to those who did not; (**E**) Kaplan–Meier curve of PFS comparing patients who developed rash to those who did not; (**F**) Kaplan–Meier curve of PFS comparing patients who developed neutropenia to those who did not; (**G**) Kaplan–Meier curve of PFS comparing patients who developed ALT increase to those who did not; (**H**) Kaplan–Meier curve of PFS comparing patients who developed hypokalemia to those who did not; (**I**) Kaplan–Meier curve of PFS comparing patients who developed hyponatremia to those who did not; and (**J**) Kaplan–Meier curve of PFS comparing patients who developed other imAEs to those who did not.

In exploratory univariable analyses of PFS, imAEs overall (HR 0.70, 95% CI 0.61–0.82; *p* < 0.0001), hyperthyroidism (HR 0.68, 95% CI 0.47–0.97; *p* = 0.03), hypothyroidism (HR 0.72, 95% CI 0.55–0.94; *p* = 0.01), and other imAEs (HR 0.65, 95% CI 0.52–0.80; *p* = 0.0001), were associated with better PFS. Neutropenia (HR 0.78, 95% CI 0.69–0.89; *p* = 0.0002) and leukopenia (HR 0.85, 95% CI 0.74–0.98; *p* = 0.02) were also associated with better PFS, whereas ALT elevation (HR 1.22, 95% CI 1.05–1.43; *p* = 0.012), decreased appetite (HR 1.42, 95% CI 1.21–1.66; *p* < 0.0001), hypokalemia (HR 1.48, 95% CI 1.13–1.95; *p* = 0.005), and hyponatremia (HR 1.54, 95% CI 1.19–1.98; *p* = 0.0008) were associated with worse PFS (Figure 4).

In the first exploratory AE-focused multivariable Cox model, which included overall imAEs and non-imAEs associated with outcome in univariable analysis, overall imAEs (HR 0.71, 95% CI 0.58–0.86; *p* = 0.0007), hyponatremia (HR 1.33, 95% CI 1.01–1.74; *p* = 0.04), decreased appetite (HR 1.46, 95% CI 1.22–1.75; *p* < 0.0001), and neutropenia (HR 0.69, 95% CI 0.58–0.81; *p* < 0.0001) were associated with OS. Overall imAEs (HR 0.66, 95% CI 0.56–0.78; *p* < 0.0001), decreased appetite (HR 1.32, 95% CI 1.14–1.54; *p* = 0.0002), hyponatremia (HR 1.27, 95% CI 1.01–1.61; *p* = 0.04), ALT elevation (HR 1.19, 95% CI 1.02–1.39; *p* = 0.03), and neutropenia (HR 0.76, 95% CI 0.67–0.86; *p* < 0.0001) were associated with PFS (Table 5).

In the second exploratory AE-focused multivariable model, in which individual imAEs and non-imAEs were entered as distinct covariates, decreased appetite (HR 1.50, 95% CI 1.26–1.80; *p* < 0.0001), hyponatremia (HR 1.39, 95% CI 1.06–1.83; *p* = 0.02), neutropenia (HR 0.67, 95% CI 0.57–0.79; *p* < 0.0001), hyperthyroidism (HR 0.37, 95% CI 0.19–0.71; *p* = 0.0035), and other imAEs (HR 0.55, 95% CI 0.42–0.72; *p* < 0.0001) were associated with OS. Decreased appetite (HR 1.37, 95% CI 1.18–1.60; *p* < 0.0001), hyponatremia (HR 1.34, 95% CI 1.06–1.69; *p* = 0.02), ALT elevation (HR 1.17, 95% CI 1.01–1.37; *p* = 0.04), neutropenia (HR 0.74, 95% CI 0.65–0.85; *p* < 0.0001), hypothyroidism (HR 0.72, 95% CI 0.53–1.00; *p* = 0.047), and hyperthyroidism (HR 0.59, 95% CI 0.37–0.94; *p* = 0.03) were associated with PFS (Table 6).

To address confounding by baseline prognosis, a complete-case Cox model was fitted, including any imAE together with age, sex, primary tumor location, prior surgery, ECOG performance status, disease stage, albumin, bilirubin, and NLR. Among 920 patients with complete data for OS (399 deaths), any imAE remained associated with a lower risk of death (adjusted HR 0.72, 95% CI 0.57–0.92; *p* = 0.008). For PFS, 918 patients (610 events) were evaluable and any imAE remained associated with a lower risk of progression or death (adjusted HR 0.63, 95% CI 0.52–0.77; *p* < 0.001) (Table 7).

Results were similar when robust standard errors were clustered by treatment center (OS HR 0.72, 95% CI 0.56–0.92; *p* = 0.009; PFS HR 0.63, 95% CI 0.49–0.81; *p* < 0.001). In an exploratory baseline-adjusted model of individual imAEs, other imAEs remained associated with OS (HR 0.39, 95% CI 0.24–0.62; *p* < 0.001) and PFS (HR 0.56, 95% CI 0.40–0.77; *p* < 0.001), and hypothyroidism remained associated with PFS (HR 0.65, 95% CI 0.43–0.97; *p* = 0.034). Rash and hyperthyroidism were not independently associated with either endpoint after full baseline adjustment.

Baseline corticosteroid exposure within 15 days before treatment initiation was recorded in 21 of 1358 patients (1.5%). Data on corticosteroid use for imAE management were available for 1276 patients (94.0%), of whom 68 (5.3%) received corticosteroids during treatment. Among exposed patients with available timing information, the median time from chemo-immunotherapy initiation to corticosteroid use was 197 days (IQR 115.5–335.3 days).

**Figure 5:**
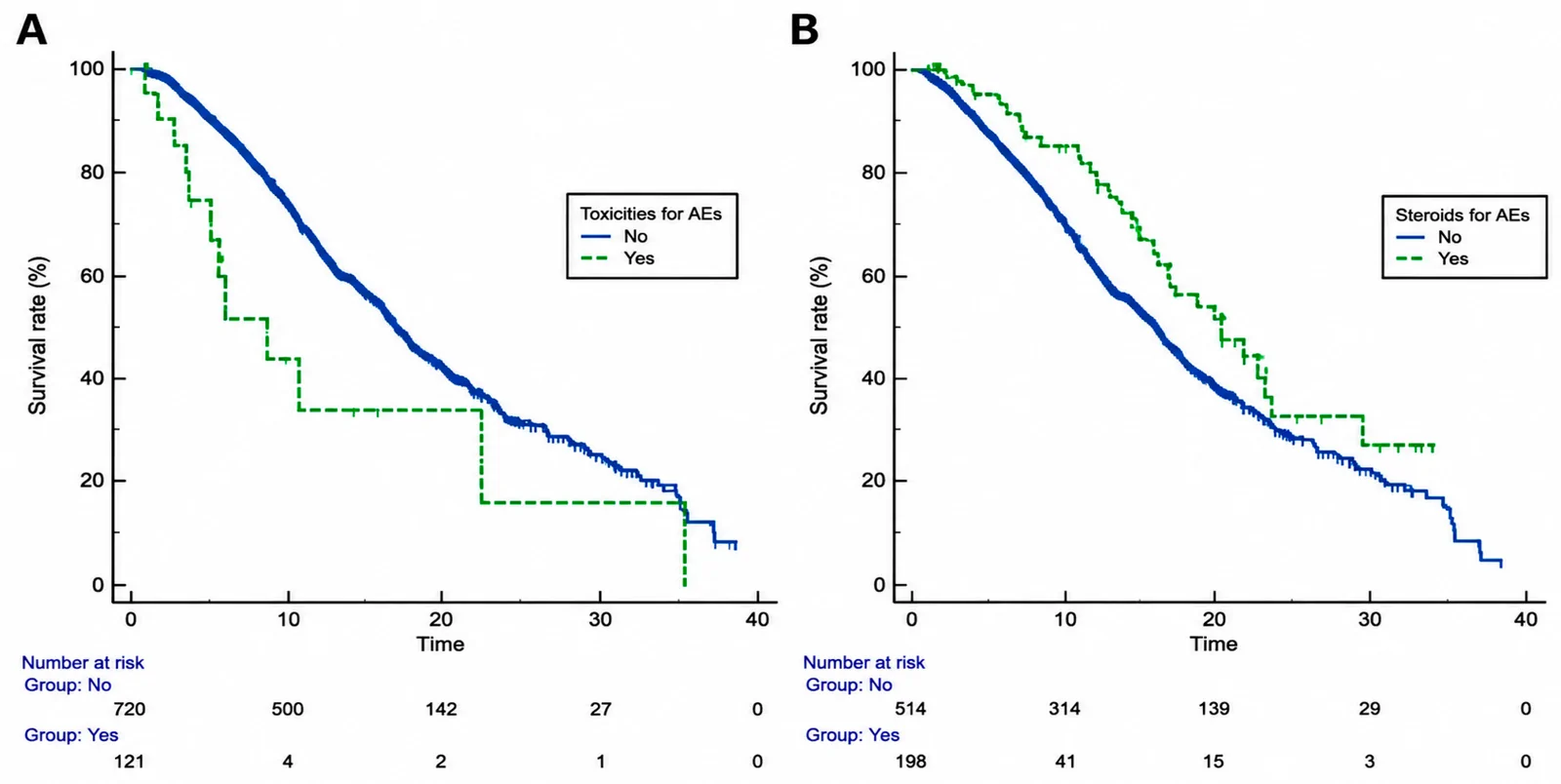
(**A**) Kaplan–Meier curve of overall survival for patients with vs. without baseline corticosteroid use within 15 days prior to treatment initiation. (**B**) Kaplan–Meier curve of overall survival for patients with vs. without corticosteroid use for immune-mediated adverse events (imAEs) during treatment.

In univariable analysis, baseline corticosteroid use was associated with shorter OS compared with no baseline corticosteroid exposure (median OS 7.7 vs. 15.6 months; HR 1.59, 95% CI 1.07–2.37; *p* = 0.021) (Figure 5A). In the original exploratory adjusted analysis including ECOG performance status, disease stage, prior surgery, albumin, bilirubin, NLR, CA19-9, CEA, and AST, the association remained statistically significant (adjusted HR 1.43, 95% CI 1.02–1.88; *p* = 0.049). Given that only 21 patients were exposed at baseline, this estimate is imprecise and highly susceptible to residual confounding and confounding by indication.

Corticosteroid use for imAE management during treatment was associated with numerically longer OS, but the difference was not statistically significant (median OS 18.6 vs. 15.1 months; HR 0.77, 95% CI 0.58–1.03; *p* = 0.071) (Figure 5B). In an exploratory model adjusted for the same baseline factors, the association remained non-significant (adjusted HR 0.79, 95% CI 0.61–1.06; *p* = 0.08).

## 4. Discussion

To the best of our knowledge, this is the first large, multicenter, international, real-world study evaluating the association of imAEs and non-imAEs with OS and PFS in patients with unresectable, locally advanced, or metastatic BTC treated uniformly with first-line cisplatin–gemcitabine plus durvalumab.

The occurrence of imAEs has been associated with favorable outcomes across several cancers treated with immune checkpoint inhibitors [28,29]. BTC-specific randomized evidence is limited but directionally consistent: an exploratory TOPAZ-1 analysis reported imAEs in 13.9% of participants receiving durvalumab plus GemCis versus 4.7% receiving placebo plus GemCis; the OS HR for durvalumab versus placebo was 0.59 (95% CI 0.30–1.23) among participants with imAEs and 0.83 (95% CI 0.70–1.00) among those without recorded imAEs [24]. Because this was a post hoc subgroup analysis, it supports biological plausibility but does not establish imAEs as a validated predictive or surrogate biomarker.

In our cohort, 276 of 1358 patients (20.3%) developed at least one imAE. Importantly, the association of any imAE with better OS and PFS persisted in a model adjusting for major baseline prognostic factors (OS adjusted HR 0.72; PFS adjusted HR 0.63) and was essentially unchanged when standard errors were clustered by treatment center. By contrast, individual-toxicity signals attenuated after full baseline adjustment: other imAEs remained associated with both endpoints and hypothyroidism with PFS, whereas rash and hyperthyroidism were no longer independently associated. AE-focused multivariable models describing patterns among toxicities should be considered exploratory rather than evidence that individual AEs are independent predictive biomarkers.

The observation that imAEs were associated with improved outcomes is biologically compatible with the hypothesis that immune activation underlying toxicity may accompany antitumor activity. Previous studies have shown that increased T-cell clonality within the tumor microenvironment can correlate with response to anti-PD-1 therapy. Since immune checkpoint inhibitors modulate T-cell function, local antitumor T-cell responses may contribute to therapeutic efficacy, while systemic T-cell activation against self-antigens may promote imAEs [30]. Nevertheless, the present observational data cannot determine whether imAEs themselves identify treatment sensitivity or simply occur more often in patients who remain on therapy long enough to develop them.

The univariable and AE-focused signals observed for thyroid dysfunction are consistent with previous reports describing endocrine imAEs as potential markers of checkpoint-inhibitor activity [31,32,33]. However, after adjustment for baseline prognostic factors in our cohort, only hypothyroidism remained associated with PFS, while hyperthyroidism was not independently associated with either endpoint. The small number of thyroid events and the large number of exploratory comparisons further limit inferences. Thyroid function monitoring remains essential for toxicity management, but any prognostic interpretation of thyroid imAEs should be considered hypothesis-generating.

Neutropenia was associated with improved OS and PFS in the exploratory AE-focused models, although its biological interpretation depends on treatment context and severity. Treatment-induced neutropenia may correlate with reduced systemic inflammatory indices such as NLR and can also reflect greater cytotoxic drug exposure. Accordingly, neutropenia may act as a clinical correlate of drug exposure or host–treatment interaction rather than a surrogate endpoint of efficacy [34,35].

In both exploratory AE-focused multivariable analyses, decreased appetite and hyponatremia were associated with poorer OS and PFS, whereas ALT elevation was associated with worse PFS. Hyponatremia has been recognized as an adverse prognostic factor across several malignancies [36,37,38,39,40]. Malnutrition and hyponatremia are closely interconnected, and decreased appetite may contribute to electrolyte imbalance; however, appetite is a subjective clinical symptom prone to underreporting in retrospective cohorts, whereas hyponatremia is laboratory-confirmed. These findings, therefore, require external validation and should not be interpreted as treatment-specific predictive effects.

Previous studies have suggested that low-grade hepatotoxicity may accompany immune activation [41]. In our cohort, however, ALT elevation was associated with worse PFS in exploratory analyses and was not significantly associated with OS after multivariable modeling. Because ALT elevation was not analyzed according to timing, etiology, or detailed toxicity trajectory, and because treatment interruption or immunosuppression may mediate its clinical consequences, the direction of this association should be interpreted cautiously.

Baseline corticosteroid use before treatment initiation was recorded in only 21 patients. Although shorter OS was observed in this subgroup, the very small exposed sample and heterogeneous indications make confounding by indication a major concern. A separate report from the same international program similarly reported worse outcomes with baseline corticosteroid exposure [42], and analogous associations have been described in other tumor types [43,44,45]. These observations are biologically plausible because corticosteroids can suppress T-cell activation and other immune functions, but the present study cannot distinguish a direct pharmacologic effect from the adverse prognosis of the conditions requiring corticosteroids. Therefore, baseline corticosteroid exposure should be regarded here as an exploratory clinical correlate, not a causal determinant of reduced immunotherapy efficacy.

Corticosteroids administered for imAE management were not significantly associated with worse OS in the exploratory analysis. This is clinically reassuring, but the result should not be overinterpreted: steroid exposure occurred after treatment initiation, was determined by toxicity occurrence and severity, and involved a relatively small subgroup. The apparent numerical survival advantage may therefore reflect the favorable association of imAEs themselves, treatment duration, and selection factors, rather than a beneficial effect of corticosteroids.

This interpretation is consistent with the literature suggesting that outcomes with corticosteroids depend strongly on treatment indication [46,47]. Steroids prescribed for cancer-related supportive care often identify patients with aggressive disease and poor prognosis, whereas steroids used to manage imAEs have not consistently been associated with inferior checkpoint-inhibitor outcomes. Clinically indicated corticosteroids should therefore not be withheld solely because of concern about immunotherapy efficacy; however, our retrospective data do not establish equivalence or safety with respect to anticancer effectiveness.

This study has several limitations. First, its retrospective design and inter-center variability may introduce selection, ascertainment, and supportive-care confounding. PFS was assessed locally without centralized radiologic review. Second, exact onset dates for imAEs and non-imAEs were not consistently captured in the harmonized database. Consequently, a reliable time-dependent AE exposure model could not be performed, and immortal time bias remains an important limitation that may inflate favorable associations for treatment-emergent toxicities. Without reliable onset dates, exposure status at fixed landmarks cannot be reconstructed consistently; residual immortal time bias therefore remains. Third, the fully baseline-adjusted model used complete cases (920 for OS and 918 for PFS), which reduced the analyzable sample; no imputation was performed. Fourth, numerous individual AEs were evaluated without multiplicity correction, so nominal borderline *p*-values are exploratory. Finally, detailed steroid dose, indication, duration, and timing were incomplete for some patients, limiting causal interpretation. Strengths include the large international cohort, uniform first-line durvalumab-based regimen, adjustment for major baseline prognostic factors, and a center-clustered sensitivity analysis.

Overall, this large international real-world cohort shows that the occurrence of any imAE during first-line cisplatin–gemcitabine plus durvalumab is associated with improved OS and PFS even after adjustment for major baseline prognostic factors. The findings support imAEs as potential treatment-emergent prognostic markers or clinical correlates of benefit, not as validated surrogate endpoints. Signals from individual toxicities, including endocrine imAEs, should be considered exploratory pending prospective confirmation with precise onset timing.

The corticosteroid findings likewise require a non-causal interpretation. Baseline exposure was uncommon and may primarily identify patients with comorbidity or poorer underlying prognosis, while post-baseline corticosteroid use for imAEs is intrinsically linked to the occurrence of toxicity and duration of treatment. Although we found no statistically significant adverse association for corticosteroids used to manage imAEs, these data should not be used to infer that timing or dose has no effect on efficacy.

Treatment-emergent clinical markers should also be viewed within the broader biomarker landscape in BTC. Molecular profiling, liquid-biopsy approaches such as ctDNA, and emerging tissue signatures may provide complementary prognostic or treatment-selection information [16,17,18]. In hepatobiliary oncology more broadly, proof-of-concept work combining extracellular-vesicle profiling with machine-learning methods illustrates how non-invasive molecular signatures may eventually be integrated with clinical variables [48]. Future BTC studies should prospectively capture exact AE onset, grade trajectory, treatment interruption, and corticosteroid timing so that time-dependent and, where assumptions are met, causal analyses can be performed alongside molecular and circulating biomarkers.

## 5. Conclusions

The occurrence of treatment-emergent imAEs was associated with longer OS and PFS in this large durvalumab-treated BTC cohort, including after adjustment for major baseline prognostic factors. These events should be considered potential prognostic markers or clinical correlates of treatment benefit rather than surrogate endpoints. Prospective validation with precise AE-onset capture and multiplicity-aware analyses is required.

## Figures and Tables

**Table 1:** Baseline characteristics (*N* = 1358).

Characteristic	Patients, *n* (%)
**Age, Years, Median**	69 (24–91)
**Sex**	
male	711 (52.4)
female	647 (47.6)
**ECOG Performance Status**	
0	684 (50.4)
1	626 (46)
2	42 (3.1)
3	6 (0.5)
**Site of the tumor**	
intrahepatic	758 (55.8)
perihilar	217 (16.1)
distal	149 (10.9)
gallbladder	234 (17.2)
**Prior Surgery**	
yes	386 (28.5)
no	972 (71.5)
**Stage of Disease**	
locally advanced	266 (19.6)
metastatic	1092 (80.4)
**Number of metastatic sites**	
<3	1076 (79)
≥3	282 (21)
**Sites of metastasis**	
liver	777 (57.2)
lymph nodes	601 (44.2)
peritoneal	336 (24.7)
lung	283 (20.8)
bone	138 (10.2)
**First-line immunotherapy**	
durvalumab	1358 (100.0)

**Table 2:** Treatment-related adverse events of any grade. Percentages for individual imAEs are calculated among the 276 patients with at least one imAE; percentages for non-imAEs are calculated using the full cohort (*N* = 1358). Patients could experience more than one event.

Adverse Event	Patients, *n* (%)
**Immune-Mediated Adverse Events**	
pruritus	112 (40.6)
rash	110 (39.9)
hypothyroidism	65 (23.6)
hyperthyroidism	27 (9.8)
colitis	21 (7.6)
other immune-mediated events	97 (35.1)
**Non–Immune-Mediated Adverse Events**	
fatigue	706 (52.0)
anemia	634 (46.7)
neutropenia	588 (43.3)
thrombocytopenia	488 (35.9)
leukopenia	419 (30.8)
thrombocytosis	141 (10.4)
nausea	408 (30.0)
decreased appetite	328 (24.1)
ALT elevation	297 (21.9)
fever	257 (18.9)
constipation	241 (17.7)
diarrhea	160 (11.8)
vomiting	159 (11.7)
neuropathy	157 (11.6)
cholangitis	125 (9.2)
hyponatremia	117 (8.6)
hypokalemia	98 (7.2)
insomnia	45 (3.3)
other non-imAEs	212 (15.6)

**Table 3:** Distribution of immune-mediated and non-immune-mediated adverse events according to toxicity grade.

Toxicity	Grade 1–2, *n*	Grade 1–2,%	Grade 3–4, *n*	Grade 3–4, %
**imAEs**	235	17.3	40	2.9
**rash**	107	7.9	3	0.2
**pruritus**	111	8.2	1	0.1
**colitis**	20	1.5	1	0.1
**hypothyroidism**	62	4.6	2	0.1
**hyperthyroidism**	27	2	0	0
**other imAEs**	71	5.2	24	1.8
**decreased appetite**	318	23.4	9	0.7
**fever**	235	17.3	22	1.6
**fatigue**	680	50.1	26	1.9
**neuropathy**	150	11	7	0.5
**nausea**	400	29.5	8	0.6
**vomiting**	152	11.2	7	0.5
**diarrhea**	148	10.9	12	0.9
**constipation**	238	17.5	3	0.2
**insomnia**	45	3.3	0	0
**cholangitis**	62	4.6	62	4.6
**anemia**	556	40.9	78	5.7
**leukopenia**	341	25.1	78	5.7
**neutropenia**	301	22.2	286	21.1
**thrombocytopenia**	386	28.4	102	7.5
**thrombocytosis**	138	10.2	3	0.2
**ALT increase**	263	19.4	34	2.5
**hypokalemia**	93	6.8	5	0.4
**hyponatremia**	102	7.5	15	1.1
**other non-imAEs**	178	13.1	32	2.4

**Table 4:** Exploratory univariable analysis of immune-mediated and non-immune-mediated adverse events. Individual-event *p*-values are nominal and were not adjusted for multiplicity.

	PFS	*p*-Value	OS	*p*-Value
	HR IC 95%		HR IC 95%	
**imAEs**				
no vs. yes	0.70 (0.61–0.82)	<0.0001	0.74 (0.62–0.89)	0.0011
**pruritus**				
no vs. yes	0.85 (0.69–1.06)	0.15	0.99 (0.76–1.32)	0.98
**rash**				
no vs. yes	0.78 (0.63–0.97)	0.03	0.69 (0.53–0.90)	0.007
**hypothyroidism**				
no vs. yes	0.72 (0.55–0.94)	0.01	0.79 (0.56–1.09)	0.16
**hyperthyroidism**				
no vs. yes	0.68 (0.47–0.97)	0.03	0.53 (0.35–0.82)	0.004
**colitis**				
no vs. yes	0.68 (0.44–1.06)	0.09	0.61 (0.34–1.09)	0.09
**other imAEs**				
no vs. yes	0.65 (0.52–0.80)	0.0001	0.60 (0.46–0.79)	0.0002
**fatigue**				
no vs. yes	1.10 (0.97–1.26)	0.14	1.20 (1.03–1.41)	0.02
**anemia**				
no vs. yes	1.02 (0.89–1.16)	0.82	0.92 (0.79–1.08)	0.32
**neutropenia**				
no vs. yes	0.78 (0.69–0.89)	0.0002	0.71 (0.60–0.83)	<0.0001
**thrombocytopenia**				
no vs. yes	0.92 (0.80 -1.05)	0.19	0.90 (0.77–1.07)	0.23
**leukopenia**				
no vs. yes	0.85 (0.74–0.98)	0.02	0.81 (0.69–0.96)	0.02
**thrombocytosis**				
no vs. yes	1.04 (0.85–1.29)	0.71	1.10 (0.85–1.43)	0.47
**nausea**				
no vs. yes	1.02 (0.89–1.18)	0.75	1.05 (0.89–1.25)	0.57
**decreased appetite**				
no vs. yes	1.42 (1.21–1.66)	<0.0001	1.58 (1.32–1.94)	<0.0001
**ALT elevation**				
no vs. yes	1.22 (1.05–1.43)	0.012	1.25 (1.03–1.51)	0.02
**fever**				
no vs. yes	1.09 (0.92–1.28)	0.32	1.09 (0.89–1.33)	0.38
**constipation**				
no vs. yes	1.02 (0.87–1.20)	0.81	1.04 (0.85–1.27)	0.72
**diarrhea**				
no vs. yes	0.97 (0.80–1.17)	0.73	0.97 (0.78–1.21)	0.78
**vomiting**				
no vs. yes	0.98 (0.80–1.19)	0.81	1.11 (0.87–1.42)	0.40
**neuropathy**				
no vs. yes	0.85 (0.71–1.02)	0.09	0.82 (0.65–1.02)	0.08
**cholangitis**				
no vs. yes	1.25 (0.99–1.56)	0.06	1.36 (1.03–1.79)	0.032
**hyponatremia**				
no vs. yes	1.54 (1.19–1.98)	0.0008	1.73 (1.28–2.33)	0.0003
**hypokalemia**				
no vs. yes	1.48 (1.13–1.95)	0.005	1.68 (1.22–2.33)	0.002
**insomnia**				
no vs. yes	0.95 (0.69–1.31)	0.74	1.23 (0.83–1.81)	0.31
**other non-imAEs**				
no vs. yes	1.11 (0.93–1.33)	0.25	1.02 (0.82–1.28)	0.85

**Table 5:** Exploratory AE-focused multivariable Cox regression models for PFS and OS including overall immune-mediated adverse events and non-immune-mediated adverse events.

	PFS HR IC 95%	*p*-Value	OS HR IC 95%	*p*-Value
**imAEs**				
no vs. yes	0.66 (0.56–0.78)	<0.0001	0.71 (0.58–0.86)	0.0007
**decreased appetite**				
no vs. yes	1.32 (1.14–1.54)	0.0002	1.46 (1.22–1.75)	<0.0001
**neutropenia**				
no vs. yes	0.76 (0.67–0.86)	<0.0001	0.69 (0.58–0.81)	<0.0001
**ALT elevation**				
no vs. yes	1.19 (1.02–1.39)	0.03	1.18 (0.98–1.43)	0.08
**hypokalemia**				
no vs. yes	1.25 (0.98–1.61)	0.07	1.28 (0.96–1.70)	0.10
**hyponatremia**				
no vs. yes	1.27 (1.01–1.61)	0.04	1.33 (1.01–1.74)	0.04

**Table 6:** Exploratory AE-focused multivariable Cox regression models for PFS and OS including individual immune-mediated and non-immune-mediated adverse events.

	PFS HR IC 95%	*p*-Value	OS HR IC 95%	*p*-Value
**rash**				
no vs. yes	0.87 (0.68–1.11)	0.26	0.74 (0.54–1.02)	0.07
**hypothyroidism**				
no vs. yes	0.72 (0.53–1.00)	0.047	0.88 (0.60–1.30)	0.53
**hyperthyroidism**				
no vs. yes	0.59 (0.37–0.94)	0.03	0.37 (0.19–0.71)	0.004
**other imAEs**				
no vs. yes	0.55 (0.42–0.72)	<0.0001	0.47 (0.32–0.67)	<0.0001
**decreased appetite**				
no vs. yes	1.37 (1.18–1.60)	<0.0001	1.50 (1.26–1.80)	<0.0001
**neutropenia**				
no vs. yes	0.74 (0.65–0.85)	<0.0001	0.67 (0.57–0.79)	<0.0001
**ALT increase**				
no vs. yes	1.17 (1.01–1.37)	0.04	1.17 (0.97–1.42)	0.10
**hypokalemia**				
no vs. yes	1.20 (0.93–1.54)	0.16	1.24 (0.93–1.67)	0.14
**hyponatremia**				
no vs. yes	1.34 (1.06–1.69)	0.02	1.39 (1.06–1.83)	0.02

**Table 7:** Fully baseline-adjusted Cox proportional hazards model for the association of any immune-mediated adverse event with PFS and OS.

Variable	Comparison	PFS Adjusted HR (95% CI); *p*-Value	OS Adjusted HR (95% CI); *p*-Value
Any imAEs	Yes vs. no	0.63 (0.52–0.77); *p* < 0.001	0.72 (0.57–0.92); *p* = 0.008
Age	Per year	0.99 (0.98–1.00); *p* = 0.008	1.00 (0.99–1.01); *p* = 0.881
Sex	Female vs. male	0.96 (0.82–1.13); *p* = 0.602	0.95 (0.78–1.16); *p* = 0.603
Primary location	iCCA vs. eCCA/GBC	1.02 (0.86–1.21); *p* = 0.804	1.02 (0.83–1.25); *p* = 0.880
Prior surgery	Yes vs. no	0.78 (0.64–0.96); *p* = 0.019	0.72 (0.56–0.94); *p* = 0.014
ECOG PS	≥1 vs. 0	1.63 (1.39–1.93); *p* < 0.001	1.97 (1.59–2.43); *p* < 0.001
Disease stage	Metastatic vs. locally advanced	1.59 (1.27–1.99); *p* < 0.001	1.70 (1.27–2.29); *p* < 0.001
Albumin	<3.5 vs. ≥3.5 g/dL	1.29 (1.06–1.56); *p* = 0.010	1.64 (1.31–2.05); *p* < 0.001
Bilirubin	Elevated vs. normal	1.17 (0.96–1.43); *p* = 0.117	1.34 (1.06–1.70); *p* = 0.014
NLR	≤3 vs. <3	1.38 (1.16–1.64); *p* < 0.001	1.65 (1.33–2.05); *p* < 0.001

Complete-case analysis: PFS N = 918 (610 events); OS N = 920 (399 deaths). No missing-data imputation was performed. Center-clustered robust-standard-error sensitivity analyses yielded similar estimates for any imAE (PFS HR 0.63, 95% CI 0.49–0.81, *p* < 0.001; OS HR 0.72, 95% CI 0.56–0.92, *p* = 0.009).

## Data Availability

The data presented in this study are available on request from the corresponding author due to privacy and ethical restrictions.

## Abbreviations

The following abbreviations are used in this manuscript:

iCCA	Intrahepatic cholangiocarcinoma
eCCA	Extrahepatic cholangiocarcinoma
GBD	Gallbladder carcinoma
OS	Overall Survival
imAEs	Immune-mediated adverse events
PFS	Progression free survival
D	Durvalumab
P	Pembrolizumab
CG	Cisplatine and Gemcitabine
BTC	Biliary tract cancer
ICI	Immune checkpoint inhibitor
HR	Hazard Ratio
